# Development and Application of Artificial Intelligence in Auxiliary TCM Diagnosis

**DOI:** 10.1155/2021/6656053

**Published:** 2021-03-06

**Authors:** Chuwen Feng, Yuming Shao, Bing Wang, Yuanyuan Qu, Qingyong Wang, Yang Li, Tiansong Yang

**Affiliations:** ^1^Heilongjiang University of Chinese Medicine, Harbin 150040, Heilongjiang, China; ^2^The First Affiliated Hospital, Heilongjiang University of Chinese Medicine, Harbin 150040, Heilongjiang, China

## Abstract

As an emerging comprehensive discipline, artificial intelligence (AI) has been widely applied in various fields, including traditional Chinese medicine (TCM), a treasure of the Chinese nation. Realizing the organic combination of AI and TCM can promote the inheritance and development of TCM. The paper summarizes the development and application of AI in auxiliary TCM diagnosis, analyzes the bottleneck of artificial intelligence in the field of auxiliary TCM diagnosis at present, and proposes a possible future direction of its development.

## 1. Introduction

AI is the main force of the fourth scientific and technological revolution [1], which is dedicated to embodying human intelligence through computational methods. It is widely used in various fields and currently mainly possesses functions such as voice and image recognition, logical reasoning ability, and emotion recognition [1, 2]. Traditional Chinese medicine (TCM) is the product of the successful combination of ancient Chinese macroscience and the medical practice of that time [3]. After thousands of years of accumulation and precipitation, a unique diagnosis and treatment system has been gradually formed, and its therapeutic effect has been widely recognized. According to TCM theory, the physiological and pathological changes of the human body's viscera and bowels, yin and yang, and qi and blood can be reflected on the outside, such as the face, tongue, pulse, and voice. Through “inspection, listening and smelling examination, inquiry, and palpation” to grasp the basic situation of the patient, the information of the four examinations is integrated to achieve the correlation of all four examinations, to realize the purpose of diagnosing diseases and symptoms. As the basis of syndrome differentiation and treatment, the four examinations are easily influenced by objective conditions such as environment and light source, as well as the subjective judgment of doctors, and lack objective and quantitative indicators. Through the combination of AI and TCM diagnosis, a large amount of TCM diagnostic information can be collected, organized, and analyzed, thus providing the possibility of establishing disease-pattern models, and promoting the objective and scientific development of TCM diagnosis.

AI with machine learning and deep learning as the mainstream ushered in a new upsurge [4]. In the 1970s, AI was applied in the field of TCM diagnosis [5], which provided a rare opportunity for the development of objective and modernized TCM diagnosis, but the problems of logical reasoning and objective quantification were not well solved and its development speed was slow [6]. In recent years, thanks to the rapid development of microsensors [7], computer image analysis, speech recognition technology [8], and deep learning [9, 10], the programmatic innovation of TCM has been accelerated and a milestone progress has been made in the standardization and normalization of TCM diagnosis [11]. The purpose of this paper is to review the application and development of AI in assisting TCM diagnosis and to analyze the current development bottlenecks and future development directions of AI-assisted TCM diagnosis.

## 2. Application of AI in the Field of Medicine

The advent of computers in the 1940s prompted people to explore the use of computers to replace or extend part of human mental work [4]. The concept of artificial neural networks was first introduced by Donald Hebb in 1949 [12], marking the nascent stage of AI. The term artificial intelligence was first coined by McCarthy in 1955 when AI was defined as the science and engineering of making intelligent machines [13]. Over the years, the term has been overused to include various computerized automated systems, logic programming, probabilistic algorithms, and remotely controlled robots [13]. It is worth mentioning that convolutional neural networks (CNN), an important part of deep learning (DL), have extensive application in various subfields of medical image analysis, including classification, detection, and segmentation. For example, the TCM facial diagnostic detector and tongue manifestation analysis system mentioned below both benefit from the development of machine learning and neural networks, while machine learning-based speech recognition technology plays an important role in listening examination.

In the 1970s and 1980s, Stanford University's Experimental Computer Research Program in Medicine was established [14], and medical expert systems were broadly used in clinical diagnosis [15], laying the foundation for the development of AI in the medical field. Nowadays, AI is widely used in modern medicine for both clinical diagnosis of common diseases [16] and image-assisted diagnosis [17, 18]. Hirasawa et al. [19] designed a CNN system capable of automatically identifying gastric cancer from a large number of endoscopic images, which is promising in aiding the diagnosis of gastric cancer. Numerous studies have shown that DL systems have a powerful ability to diagnose diabetic retinopathy [20]. And some achievements have been made in the application of auxiliary surgery [21], the most representative being the da Vinci surgical robot [22]. Multiple pieces of evidence support that AI and big data classifiers play a vital role in the field of organ transplantation [23]. A group of researchers have proposed a facial analysis framework for genetic syndrome classification, called DeepGestalt, which uses computer vision and DL techniques to quantify the similarity of hundreds of symptoms. The accuracy of the test of identifying correct symptoms on 502 different images was 91%, which was better than that of clinicians. DeepGestalt has potential application value in the phenotypic evaluation of clinical genetics and gene testing [24]. The application of artificial intelligence nursing providers (AICP) in the field of mental health care and treatment is also an embodiment of the development of AI virtual technology [25].

The combination of TCM and AI began in the 1970s, with the birth of the first international expert system of TCM, which was named “TCM Guan Youbo Hepatitis Diagnostic and Treatment Procedures,” as a landmark event. In the decade since, AI has also made progress in TCM disease diagnosis, assisting in standardizing TCM diagnostic models [26]. Recently, Zhang et al. [27] proposed an AI-based TCM auxiliary diagnosis system, which can diagnose 187 common TCM diseases and related syndromes. The prediction accuracy of the top 3 and the top 5 disease types were 80.5%, 91.6%, and 94.2%, respectively, indicating that they had precise diagnostic accuracy. Besides, AI has an active impact on the development and quality monitoring of new Chinese medicines, the construction of Chinese medicinal prescription models, and acupuncture point combination [28, 29]. Yao et al. [30] proposed an ontology-based artificial intelligence model for medicine side-effect prediction, and these predictions were validated with neural network structures, but the model is highly dependent on sufficient clinical data, and more in-depth exploration to improve the accuracy of the predictions is necessary in the future.

## 3. Application of AI in Auxiliary TCM Diagnosis

### 3.1. Inspection

As the first of the four TCM examinations, inspection has the characteristics of intuitiveness and simplicity and plays an important role in the TCM diagnosis. Through inspection, the physician observes the patient's general or local appearance and morphology, thus achieving the goal of determining the patient's disease state. The contents of TCM inspection are numerous, with particular emphasis on facial and tongue diagnosis in clinical practice.

#### 3.1.1. Facial Diagnosis

Changes in facial color and luster can reflect the glory and decline of qi and blood of the corresponding viscera and bowels and meridians. AI plays a key role in the recognition and extraction of facial information, so it has been used to study the differences and correlations of facial information between different diseases as well as between different patterns of the same disease. Dong [31] applied a TCM face digital detector to collect and analyze the facial color characteristics of patients with coronary heart disease, chronic renal failure, and chronic hepatitis B. The results showed that there were significant differences in the facial color index between the three diseases, indicating that there is a pattern of changes in the facial color and its parameters in different visceral diseases. Liu et al. [32] used a TCM face detection instrument to collect facial features of 60 patients with chronic nephritis, to study the facial features of patients with damp-heat type chronic nephritis and to analyze the relationship between the facial features of nephritis patients and changes in renal function. It was found that there were differences in facial color parameters between different types of patients, and there was a correlation between facial color parameters and renal function testing indicators. Guo et al. [33] applied the TCM face detection instrument to carry out a study on the correlation between different stages of chronic renal failure and the changes in face information and found that the face color index of patients in the decompensated stage of renal function and the uremia stage decreased significantly, which confirmed that there is a certain correlation between the disease stages of chronic renal failure and the face color parameters.

#### 3.1.2. Tongue Diagnosis

The tongue and internal viscera and bowels are connected by meridians. Exuberance and debilitation of the healthy qi or pathogenic qi and the changes of qi, blood, fluid, and humor can be obtained by observing the tongue manifestation. Tongue diagnosis mainly includes looking at the tongue body and tongue fur. The tongue body mainly reflects the patient's exuberance and debilitation of qi and blood and strength and weakness of the viscera and bowels. The location and nature of the disease can all be reflected by tongue fur. To integrate the collection, processing, and analysis of tongue manifestation into a whole procedure and make TCM tongue diagnosis tend to be more intelligent, the development and application of tongue manifestation analyzer and “TCM tongue diagnosis automatic identification system” have been set off.

As early as the 1990s, the “TCM tongue diagnosis automatic identification system” established by Tsinghua University and Xiyuan Hospital of China Academy of Traditional Chinese Medicine realized the quantitative analysis of tongue body and tongue fur. In an experiment using this system to observe the tongue body of patients with blood stasis, the analysis result was 86.34% consistent with the visual observation [34]. At the beginning of the 21st century, Jiang et al. [35] designed a computerized tongue diagnosis system of TCM to analyze the characteristics of the tongue using fuzzy theory, which can initially read the amount of tongue fur, the bias in the distribution of the tongue fur, and the thickness of the tongue fur. Cui et al. [36] applied the “TCM tongue diagnosis expert system” to quantitatively study the tongue manifestation of patients with stroke disease, and the results were consistent with the characteristics of the mechanism of disease changes in the acute and recovery phases of stroke disease. Zhang et al. [37] designed a Bayesian network-based tongue diagnosis auxiliary system, and the accuracy of which was higher than 75% in identifying tongue manifestation of healthy individuals, patients with pulmonary heart disease, appendicitis, gastritis, pancreatitis, and bronchitis. Lo et al. [38] extracted nine tongue manifestation features such as tongue color and tongue body using an automatic tongue diagnosis system and further divided the extracted features by region to achieve screening for early breast cancer. Han et al. [39] used a tongue diagnosis information acquisition system to collect tongue manifestation from colorectal cancer patients and healthy individuals and analyzed the thickness of their tongue fur. The study showed that the tongue fur of colorectal cancer patients was significantly thicker than that of healthy people, which provided a basis for tongue diagnosis as an early screening tool for colorectal cancer.

Tongue manifestation analyzer can be adjusted according to the patients' tongue diagnostic environment and tongue spitting posture, and it can acquire the tongue in a multidimensional manner, analyze the tongue manifestation completely, evaluate the tongue accurately, and finally store data and imaging [40]. In recent years, many scholars have used tongue analyzers to study the correlation between pathological evidence and tongue manifestation and between tongue manifestation and objective manifestation indicators. Xu et al. [41] applied the TP-1 TCM tongue and pulse condition digital analyzer to study the tongue manifestation of patients with deficiency symptoms of chronic renal failure and found that the tongue color index and the moist and dryness index were of great reference significance for the differentiation, especially the tongue color index was more meaningful. Zhang et al. [42] used a DS01-B tongue information acquisition device to collect tongue color information of 273 nontraumatic femoral head necrosis patients, used frequency analysis and cluster analysis to analyze the distribution characteristics, and concluded that the tongue color indices of different ARCO (Association Research Circulation Osseous) stages have different characteristics. It provides an objective basis for TCM diagnosis of nontraumatic femoral head necrosis.

### 3.2. Listening and Smelling Examination

Listening and smelling examination is a diagnostic way for physicians to understand the various abnormal sounds and smells emitted by patients through hearing and smelling [43]. Listening to sounds includes voice, breathing, coughing, yawning, sighing, snoring, sneezing, borborigmus, and splashing sound [44]. By smelling and listening, the doctor can determine the nature and location of the disease and predict the progression and prognosis of the disease [45].

#### 3.2.1. Listening Examination

In the listening examination, AI is mainly used in physique identification, disease diagnosis, syndrome type research, and evaluation of clinical efficacy through voice information. Wang [46] collected voices of normal adults aged 20 to 79 years old, used a computer voice analyzer to obtain voice data, and concluded that the voice changes with age; also, there is a positive correlation between the voice changes and exuberance and debilitation of Qi. It provides an objective basis for TCM to understand the deficiency and excess syndromes of voice diseases of different ages. Qian et al. [47] used BD-SZ auxiliary diagnostic instrument to identify the five-phase constitution by audio characteristics of 36 Parkinson's sufferers. The results showed that there were more wood, earth, and water constitution, and less metal constitution, among which wood constitution was the most, suggesting that Parkinson's disease and wood constitution may have a certain correlation. Dong et al. [48] used the “Collection System of TCM Auscultation” to capture the speech signals of chronic pharyngitis patients and obtained the energy feature data of different frequency bands by wavelet packet decomposition method and found that there were different energy characteristics in more frequency bands between patients with chronic pharyngitis and normal people, the chronic pharyngitis lung qi deficiency group, the phlegm-heat accumulation group, and the yin deficiency and lung dryness group. Li et al. [49] collected TCM acoustic parameters of patients with bronchial asthma in remission before and after treatment by using “acoustic information acquisition system” and found that the resonance peak indexes F1 and F2 of patients with asthma in remission were significantly reduced after treatment, which provided an effective basis for the clinical efficacy evaluation of bronchial asthma.

AI has also been applied to the study of the phoneme theory of the five viscera in the listening examination. Based on the theory of “five visceral phonemes,” Chen et al. [50] used the “TCM acoustic diagnosis acquisition system” to collect sound signals from patients with lung, liver, spleen, kidney, and heart diseases as well as normal people and analyzed the sound signals using the sample entropy method. The differences in the sample entropy values of the six time-domain frequency bands were statistically significant (*P* < 0.05), which provided an objective basis for the localization of diseases in viscera and bowels based on listening examination information in TCM. Zheng [51] used a 25-tone analyzer to detect and analyze the average frequencies of the voices of women with hot and cold constitutions and found that the difference in the average number of times in the pinnacle and angular regions between cold and hot constitutions was statistically significant.

In addition, AI has also made progress in the recognition of other pathological sounds. Allwood et al. [52] described the progress of AI in signal recognition and processing of hyperactive bowel sounds, and Abeyratne et al. [53] designed an automated algorithm to diagnose pneumonia by extracting parameters from patients' cough and breath sounds.

#### 3.2.2. Smelling Examination

The application of AI in the smelling examination is mainly in the recognition and analysis of odor signals. The e-nose based on advanced array gas sensor technology can grasp the odor information from a holistic perspective [54], which provides good technical support for the objective research of TCM smelling examination. Lin et al. [55] used e-nose to accurately identify the odor map characteristics of type 2 diabetes patients and healthy individuals. Based on the principle of e-nose, Liu [56] combined the sensor technology, signal processing technology, and pattern recognition technology and optimized the artificial neural network recognition algorithm to construct an oral odor detection system for TCM diagnosis.

### 3.3. Inquiry

Inquiry is a diagnostic method for doctors to obtain complete and true information about a patient's condition through purposeful questioning of the patient and his or her family to understand the occurrence and development of the disease. The content of TCM inquiry is mainly based on the “Ten Question Song” created by Zhang Jingyue, but nowadays, it also incorporates past history, allergy history, and family history in modern medical records [57]. In the early stages of certain diseases, the lack of objective signs of abnormalities makes it important to obtain information about the patient's condition through inquiry [58].

AI facilitates the development of TCM inquiry systems. He et al. [59] combined computer technology, intelligent information processing technology, and TCM theory to try to develop a computerized TCM inquiry system. The system collects the basic information of users and the information of inquiry symptoms through the front desk module and the inquiry processing module, stores them in the inquiry database of user symptoms, and finally makes the preliminary judgment of the inquiry results through the diagnosis module based on the criteria set in the inquiry criteria database. Afterwards, they tested the system on 1767 clinical patients in internal medicine, surgery, gynecology, and pediatrics, compared the test results with the experts' interpretation, and found that the clinical interpretation rate of the system was 90%. Similarly, Zheng et al. [60], based on the TCM splenic inquiry scale, combined with TCM clinical practice, designed a standardized inquiry information collection system for TCM splenic diseases. The system has been tested by experts and clinicians and can collect systematic and standardized clinical information from patients, and the data analysis function is convenient and fast. However, there is a lack of research and evaluation on the accuracy of the diagnosis. Liu et al. [61] have developed a TCM heart disease inquiry system, which provides a complete and standardized record of inquiry information, but the results of the diagnosis are determined by clinical experts on their own.

### 3.4. Palpation

The method of palpation is for doctors to understand the health status and diagnose the patients' condition by touching and pressing on certain parts of the patient [62]. Among them, the wrist pulse-taking method is the most common and important method of palpation. The movement of qi and blood can affect the changes of the pulse condition, and the pulse condition can be used to understand the location and nature of the disease.

The application of AI in pulse diagnosis is mainly reflected in the acquisition and analysis of pulse condition information, which to some extent solves the problem that the results of pulse diagnosis are susceptible due to the lack of objectivity influenced by the sensitivity of the doctors' fingertips and clinical experience.

Among the methods of pulse condition-information analysis, time-domain analysis, frequency domain analysis, time-frequency analysis, and wavelet analysis are mainly used [63]. Yang et al. [64] applied the ZBOX-I pulse digital analyzer to study the parameters of the pulse map of the three parts of the right wrist pulse condition in patients with IgA nephropathy with spleen-kidney qi deficiency pattern by frequency domain analysis and concluded that the 24 h urine protein quantification was negatively correlated with the pulse map parameters *w*/*t* and h3/h1 and positively correlated with h1 and h5, which could provide a relevant basis for the clinical diagnosis and treatment of patients with IgA nephropathy with spleen-kidney qi deficiency pattern. Using a TP-I digital pulse analyzer, Yan et al. [65] compared the distribution of pulse shape and parameters of pulse maps of the right and left hand of 119 pregnant women with those of normal subjects by time-frequency analysis. The results showed that in the normal group, normal pulse and slippery pulse were most common; in the pregnant group, slippery pulse and string-like pulse were most common. The PSR_1_ and BF of the left and right pulses of the pregnant group were significantly higher than those of the normal group, and the LMS_2_ was significantly lower than those of the normal group. Mo and Wang [66] designed a pulse condition detection system based on wavelet analysis to judge the subhealthy population, which is of high accuracy in identifying subhealth states.

In terms of pulse condition-information acquisition, Sun [67] designed a pulse condition acquisition system based on a mobile terminal. Through comparison experiments with the standard pulse diagnosis system and validation analysis of the pulse characteristics of arteriosclerosis patients, the results showed that the system could accurately acquire pulse information of normal humans and arteriosclerosis patients. Zhang [68] designed a wearable pulse condition detection and analysis system. Jin [69] designed a portable three-position pulse condition acquisition system that can meet the needs of home healthcare and experimental research. In recent years, the development of TCM remote pulse diagnosis systems has also begun to emerge. Wang and Bai [70] designed an adjustable closed-loop remote pulse diagnosis system based on virtual reality technology. She [71] designed the TCM remote pulse diagnosis system to initially complete the acquisition and reproduction of the TCM finger technique and patients' pulse. Wu et al. [72] applied the Junlan pulse diagnosis bracelet to explore the characteristics and regularity of the pulses of healthy female college students during their menstrual cycle and found that the slippery pulse was the most common during the menstrual cycle, and the rough pulse was more common during ovulation.

## 4. Discussion

AI has great potential in the development of healthcare and presents an opportunity to modernize the development of TCM diagnostics. Over the past decades, many scientists and medical scientists have contributed to the combination of them. The research and development of intelligent TCM diagnostic instruments, the advent of TCM expert diagnostic systems, and the realization of TCM diagnosis based on cell phone platforms are all exciting research results that have greatly promoted the development of TCM diagnosis and healthcare. Combining AI with TCM diagnosis cleverly avoids the malpractice of uncertainty in doctors' subjective judgment, makes the diagnosis information more real, and improves the accuracy of clinical diagnosis. The application of AI in the early screening and diagnosis of certain diseases will facilitate early understanding of the disease and halt its progression.

Although AI has made some achievements in the application of TCM diagnosis, there is still a lot of room for development. For AI diagnostic accuracy in auxiliary TCM diagnosis, there seems to be a lack of relevant reports. For inspection, the application of AI is limited only to facial and tongue diagnosis, and inspection has not been given to other parts of human body. Although objective studies of tongue color, tongue body, and tongue fur have been made in tongue diagnosis, there remain certain difficulties in the study of the motility of the tongue. The tongue color is easily influenced by food and medication, and it is worthwhile to explore how to intelligently identify whether it is influenced by such factors. In the facial diagnosis, it is mainly limited to the analysis of facial color. Although there are studies on the analysis of facial expressions in some countries, it is incapable of establishing a connection between facial changes and TCM symptoms. As to facial color analysis, how to intelligently determine the true nature of the disease, for instance, whether the redness of the face is caused by heat pattern or a pattern of exuberant yin repelling yang, is also a question that will need to be addressed later. The role of AI in the listening examination is mainly to study the speech sounds of different diseases, but the study is limited to lung, vocal cord, and throat diseases, while the study of speech sounds of other clinical diseases and the study of other pathological sounds such as vomiting sounds are less reported. The application of AI in the smelling examination is based on the study of gas composition and the oral odor by electronic nose technology, but it has not been possible to determine the corresponding TCM syndrome and disease by smelling the odor. In the inquiry, human-computer dialogues can be used to obtain information about diseases, which can avoid the problem of emotional stress that affects the presentation of medical information in some patients when facing doctors. However, there are still problems such as the language of inquiry is not standardized and the inquiry of complex diseases is not yet realized. Based on TCM thinking, the development and research of inquiry system programs should also integrate the consultation contents of modern medicine, including past history and family history, to realize the combination of Chinese and Western medicine diagnosis. With the help of AI, experts and scholars need to develop an inquiry system that reflects humanistic care and adopts easy-to-understand language for the elderly and children, which is the direction that experts and scholars need to work on in the future. In recent years, the emergence of remote pulse diagnosis systems, pulse diagnosis bracelets, and wearable pulse diagnosis systems has provided patients with the convenience of remote pulse diagnosis, but more technical support is needed to develop a simulator that can reproduce a completely real pulse condition. Also, there is a saying in TCM theory that “correspondence between nature and human” and the change of pulse condition is related to the seasons and natural climate; for example, spring is dominated by string-like pulse and winter is dominated by sunken pulse.

Over the past decades, a large number of TCM expert diagnostic systems have been developed, but due to the complexity of TCM information, the lack of unified diagnostic criteria, and the inflexibility of the systems, there are still some shortcomings in applying them to clinical diagnosis. Therefore, building a complete and true four-diagnosis information database, establishing international standardized diagnostic standards, and using the application advantages of deep learning algorithms such as CNN, recurrent neural network (RNN), and deep neural network (DNN) in the field of medicine, developing a smarter TCM diagnosis system is a key direction that experts in AI and TCM will need to work on in the future.

The intelligence of TCM diagnosis will be the path to the early modernization of TCM. For AI to be universally applied in clinical diagnosis, not only do we need to train multidisciplinary crossover talents and complete the development of relevant intelligent devices with certain financial support. Secondly, the state needs to improve relevant policies and regulations to protect patients' personal data and disease-related information from being leaked and applied for other purposes. In addition, a reasonable cost standard for the use of smart diagnostic devices should be set. With the innovation of AI programming and data, there will be more breakthroughs in AI in the field of TCM diagnosis, and the day of realizing diagnosis at home is just around the corner. With the help of AI, TCM diagnosis will certainly be more accurate and convenient in the future and will play an important role in promoting the development of medical practice worldwide.

## Data Availability

This study has no laboratory data, but the review study process and reference records are corrected and put in the data center of Heilongjiang University of Chinese Medicine, and these data will be kept for 8 years.
